# Electrostatic Modes of Application, Therapeutics and the Uses of the Roentgen Ray

**Published:** 1904-01

**Authors:** 


Electrostatic Modes of Application, Therapeutics and the Uses of the Roentgen Ray. By William Benham Snow, M. D., Professor of Electrotherapeutics and Radiotherapy in the New York School of Physical Therapeutics. Second edition revised and enlarged. Over one hundred illustrations, including ten full page half-tones. A. L. Chatterton & Co. New York. 1903. (Price, $3.00.)

The initial portion of the book is technical. After a reference to the growing interest concerning electrostatic modes of application in many forms of disease, the author describes the various kinds of apparatus now before the public, and gives directions as to the care of the machines, especially during periods of humidity. This latter point he elaborates in an unusual degree.

The volume contains valuable hints, growing out of the experience of the writer, upon a subject that so perplexes the general practitioner. Physics constitutes a most important feature in the general principles of electrostatic administration, and due attention has been paid by the author to this branch of his subject. Information upon this point is strenuously demanded, since many physicians are today employing the system without anything like a proper knowledge of the principles underlying the inevitable effects of its practical use. There is a short chapter concerning *x*-ray apparatus, setting forth the respective merits of the static machine and the Rumkorff coil. It was a good thought to place in opposing columns their advantages and disadvantages. The preference of Dr. Snow for the static machine is too strongly emphasised, since the coil, too, has its splendid advantages.

The therapeutic application of the static machine is carefully discussed with special reference to passive hyperemia, sprains, all forms of nervous conditions and paralysis,—in fact, to a great many diseases. There is no doubt in the author's mind that under certain conditions, a temporary relief is secured by the use of the static machine, although he certainly over-estimates its influence.

Snow also considers the different classes of disease in which this new method has been made the subject of experiment, and those in which its value has been actually demonstrated. There is no doubt that the *x*-ray has more effect superficially than upon deep-seated conditions. In spite of the great power of penetration it is not as likely to relieve these conditions as those merely cutaneous; nor is it infallible even there. If this is true, its power upon deep-seated disease is limited. There are, of course, diseases which have not been amenable to treatment and which still demand investigation. We can heartily commend the work, containing as it does a fair and reasonably full summary of the best upon a most important subject. The mechanical features of the book are commendable and the illustrations are fine.

